# Work and Mental Complaints: Are Response Outcome Expectancies More Important Than Work Conditions and Number of Subjective Health Complaints?

**DOI:** 10.1007/s10926-016-9648-z

**Published:** 2016-06-24

**Authors:** Tone Langjordet Johnsen, Aage Indahl, Hege Randi Eriksen, Camilla Ihlebæk, Torill Helene Tveito

**Affiliations:** 1 Division of Physical Medicine and Rehabilitation, Vestfold Hospital Trust, POB 2168, 3103 Tønsberg, Norway; 2 Uni Research Health, POB 7810, 5020 Bergen, Norway; 3 Department of Health Promotion and Development, University of Bergen, Bergen, Norway; 4 Section of Public Health, ILP, Norwegian University of Life Sciences, Ås, Norway; 5 Department of Sport and Physical Activity, Bergen University College, Bergen, Norway; 6 Department of Health Promotion, University College of Southeast Norway, Horten, Norway

**Keywords:** Subjective health complaints, Anxiety, Depression, Occupational health, Coping

## Abstract

*Purpose* Investigate the relative effect of response outcome expectancies, work conditions, and number of subjective health complaints (SHC) on anxiety and depression in Norwegian employees. Learned response outcome expectancies are important contributors to health. Individual differences in the expectancy to cope with workplace and general life demands may be important for how work conditions influence health. *Method* A survey was conducted among 1746 municipal employees (mean age 44.1, SD = 11.5, 81.5 % female), as part of a randomized controlled trial. This cross-sectional study used baseline data. Multiple logistic regression analysis was performed. Outcome variables were anxiety and depression; response outcome expectancies, work conditions, and number of SHC were independent variables. *Results* A high number of SHC was a significant factor in explaining anxiety (OR 1.26), depression (OR 1.22) and comorbid anxiety and depression (OR 1.31). A high degree of no and/or negative response outcome expectancies was a significant factor in explaining depression (OR 1.19) and comorbid anxiety and depression (OR 1.28). The variance accounted for in the full models was 14 % for anxiety, 23 % for depression, and 41 % for comorbid anxiety and depression. *Conclusion* A high number of SHC, and a high degree of no and/or negative response outcome expectancies were associated with anxiety and depression. The strongest association was found for number of SHC. However, previous studies indicate that it may not be possible to prevent the occurrence of SHC. We suggest that workplace interventions targeting anxiety and depression could focus on influencing and altering employees’ response outcome expectancies.

## Introduction

Subjective health complaints (SHC) are general health problems with a high prevalence, affecting more than 90 % of the general population in Norway [1, 2]. SHC refers to somatic and psychological complaints without objective pathological signs or symptoms, or where the pathological findings are disproportionate to the illness experience [3]. Anxiety and depression are common psychological complaints, affecting 20–25 % of the adult population (see e.g. 4, 5).

Anxiety and depression has emerged as a major public and occupational health problem in many countries [6]. Depression and mild anxiety disorders are the most common mental disorders among employees, with a prevalence of between 6 and 10 % on a subclinical level (see e.g. 6, 7). As with other mental disorders, the core symptoms of anxiety and depression affect a person’s emotional, cognitive and social functioning, which can have impact on working ability [8]. Studies based on records of sick leave certificates indicate that employees diagnosed with anxiety or depression often show a pattern with long duration and frequent recurrence of sick leave [9], and multiple episodes of sick leave is a risk factor for permanent exclusion from working life [10]. People who are employed have significantly better health compared with those who are outside the labour market [11], and being on disability benefits is a risk factor for early death [12]. The increase in sick leave and work disability because of anxiety and depression has serious negative health and economical consequences and thus calling for preventive strategies [13].

As the activity occupying most people’s waking time is work, the work environment is an important arena for influencing the health of employees. Unemployment is a more important determinant for poor mental health than work-related risks, but in those who are working, the perception of high demands, low control, and high strain, as proposed in the ‘job strain’ model [14], and low work satisfaction are significantly associated with increased risk of anxiety and depression [15, 16]. Coping is also an important factor influencing the mental health of employees, as prolonged stress activation as a result of lack of coping might lead to a feeling of helplessness and hopelessness, and both of these conditions are proposed as cognitive models of depression [17, 18]. Coping increases resistance to development of mental disorders (see e.g. 19), and has been shown to be more important for health than control [20].

Coping is defined and measured in many different ways. The ‘transactional model of stress and coping’, which focuses on coping strategies [21], and self-efficacy, which focuses on the belief that a person can act in a way that leads to a particular goal [22], are influential models. However, in this study, coping is defined and measured as a positive response outcome expectancy, based on the Cognitive Activation Theory of Stress (CATS) [18]. CATS offer a psychobiological explanation for the presumed relationships between health and internal and external events. These events are referred to as “stress” [18]. Whether an event is pleasant or threatening depends on a person’s appraisal of the situation, which again is based on previous experience and learning and expectations of one’s responses [18]. Specific responses or coping strategies may alter the stress stimuli, and these effects will be stored as response outcome expectancies. CATS states that the strategy chosen does not predict a person’s internal state and thus it does not predict health effects [18]. CATS argues that coping predicts relations to health and disease only when it is defined as positive response outcome expectancy, and that the most important aspect of coping for health outcomes is not how a person copes but rather if a person expects to cope at all [18]. In CATS, response outcome expectancies may be positive (coping), negative (hopelessness), or the individual may have established no response outcome expectancy (helplessness). The ability to react to challenges and changes with a general alarm response is an essential element of our self-regulating system. The alarm response elicits a general increase in wakefulness and brain activation, and specific responses to manage the reason for the alarm [18]. But, there is no linear relationship between the challenges or demands the individual is faced with, and the increase in activation. It is the individual’s experience of the demands and the expectancies of the response outcome that is important for the duration of the activation. A short-lasting activation has no proven ill effects, but may rather have a positive training effect [18]. Long-lasting or sustained activation may however produce negative health effects, illness or disease [18]. Individual differences in the expectancy and ability to cope with workplace and general life demands may thus be important for how the work conditions influence the health of the employees [19, 20].

Somatic and mental complaints are frequently co-occurring. Unexplained or multiple somatic symptoms are strongly associated with coexisting depressive and anxiety disorders (see e.g. 23, 24), and the prevalence rates of mental disorders is found to increase with the growing number of somatic disorders [25]. Anxiety and depression are also often co-occurring, and 85 % of adults with depression experience significant symptoms of anxiety, and 58 % have a diagnosable anxiety disorder during their lifetime [26, 27]. However, it is important to remember that there are many similarities between anxiety and depression in terms of risk factors, symptoms, and genetic factors [28]. In general, there is a strong association between number of symptoms and overall health and functional status, and the simple method of counting symptoms might be valuable in research on medically unexplained conditions [29, 30].

The aim of this study was to explore the association between employees reporting anxiety and/or depression on the Subjective Health Complaint inventory (SHC), a inventory that records complaints, without asking for attributions or medical diagnosis [31], and response outcome expectancies, work satisfaction, physical and mental work strain, and number of SHC. We hypothesize that response outcome expectancies is a stronger predictor for anxiety and depression than work satisfaction, physical and mental work strain and number of SHC.

## Method

### Sample and Procedure

The sample consisted of 1746 Norwegian municipal employees recruited from two municipalities in Norway, as part of a large randomized controlled trial; ‘at Work’ [32]. All municipal employees above 18 years of age in the cities of Kongsberg and Horten, Norway, were invited to participate in the study. At the start of the study, it was estimated to be approximately 1500 municipal employees in Kongsberg and 2000 in Horten, giving a response rate of approximately 50 %. 1716 employees answered the item regarding anxiety, and 1721 employees answered the item regarding depression; 24 employees did not answer the anxiety nor the depression item and were excluded from the analysis, leaving a total sample of 1722 employees [81 % females, mean age = 44.1, SD = 11.5, mean years of education 14.5 (SD = 3)].

### Ethical Considerations

The study was conducted according to the Declaration of Helsinki [33], and was approved by the appropriate ethics committee (REK-vest, ID 6.2008.117), and data protection officials (NSD, ID 18,997, Rikshospitalet, ID 08/2421). A declaration of informed consent was collected from all participants.

### Instruments

#### Outcome Variables

*Anxiety and depression* were measured by the Subjective Health Complaint inventory (SHC) [31]. SHC is a reliable and valid measure of common health complaints [31] and consists of 29 questions concerning subjective somatic and psychological complaints experienced during the last 30 days. The SHC inventory records complaints, without asking for attributions or medical diagnosis [31]. The selection of questions is based on frequent health complaints and reasons for encounter with the general practitioner, and is not based on any specific theory [3]. The severity of the complaints is rated on a four point scale (0~“not at all”, 1~“a little”, 2~“some”, 3~“severe”). The SHC inventory yields five subscales: musculoskeletal complaints (headache, neck pain, upper back pain, low back pain, arm pain, shoulder pain, migraine, and leg pain during physical activity), pseudoneurology (extra heartbeats, heat flushes, sleep problems, tiredness, dizziness, anxiety, and sadness/depression), gastrointestinal problems (heartburn, stomach discomfort, ulcer/non-ulcer dyspepsia, stomach pain, gas discomfort, diarrhea, and obstipation), allergy (asthma, breathing difficulties, eczema, allergy, and chest pain), and flu (cold/flu and coughing). In this study we used the items measuring anxiety and depression in the SHC inventory as outcome variables. The exact wording of the anxiety and depression items on the SHC was “anxiety” for the anxiety item and “sad, depressed” for the depression item. These two single items in SHC is found to perform similar with two widely used and validated questionnaires, The Hospital Anxiety and Depression Scale (HADS) and Hopkins Symptom Checklist–25 (HSCL), in identifying anxiety and depression [34]. Employees were regarded to have substantial complaints if they had answered some (score 2) or severe (score 3) in answer to “degree” on the anxiety and depression items in SHC [1].

#### Predictor Variables

*Response outcome expectancy* was measured by nine items from The Theoretically Originated Measure of the Cognitive Activation Theory of Stress (TOMCATS) [35]. It is a newly developed scale, designed to measure response outcome expectancies as defined in CATS [18]. The scale consists of three factors, which represent the three response outcome expectancies in CATS: positive expectancy (coping) (two items), no expectancy (helplessness) (four items) and negative expectancy (hopelessness) (three items). The three factors consists of the following statements: (1) Coping: “When I prioritize a task, I usually achieve my goal” (#1) and “I can solve most difficult situations with a good result” (#7) (α = 0.5), (2) Helplessness: “Experience has taught me that even big attempts gives very small results” (#9), “I really don’t have any control over the most important issues in my life” (#4), “All my attempts at changing my life are meaningless” (#8), and “I wish I could change my life, but it’s not possible” (#6), (3) Hopelessness: “All my attempts at making things better just make them worse” (#2), “It’s better that others try to solve my problems than for me to mess things up and make them worse” (#5), “I would have been better off if I didn’t try so hard to solve my problems” (#3). All items were rated on a five point scale from 0~”not true at all”—4~“completely true”. In a previous study of a Swedish population [35], the inventory proved to have high reliability and a clear factor structure. In this study helplessness and hopelessness are treated as one factor due to the results on factor and reliability analysis [36]. Chronbach’s alpha of the helplessness/hopelessness construct was 0.79.

*Work satisfaction* was measured by two single questions: “Do you enjoy your work?”, with the response categories; 0~“no”, 1~“sometimes”, 2~“yes”, and “How satisfied are you with your work when you take into consideration the work routines, management, salary, opportunity for advancement and work colleagues?”, rated on an eleven point scale ranging from 0~“not satisfied” to 10~“very satisfied”.

*Physical and mental work strain* was measured by two single questions: “Do you have heavy/repetitive work?”, rated on an eleven point scale ranging from 0~“not at all” to 10~“very heavy/repetitive”, and “Do you experience your current work as stressful?”, rated on an eleven point scale ranging from 0~“not stressful at all” to 10~“very stressful”.

*Number of substantial subjective health complaints* was measured by the 27 remaining items of the Subjective Health Complaint inventory (SHC) [31]. We used the method of counting symptoms, as proposed by Kamaleri et al. [30]. Like the outcome variables, employees were categorized to “substantial complaints” if they responded “some” (score 2) or “severe” (score 3) on “degree” of SHC [1].

### Statistics

All analyses were conducted using SPSS version 16.0 (Chicago: SPSS Inc). Our models contained ten independent variables used to assess the likelihood that respondents would report anxiety and/or depression, or comorbid anxiety and depression in the last 30 days. The outcome variables were dichotomized to 0~“not at all” or “a little”, and 1~“some” or “severe”, and logistic regression analyses were used to test the study hypothesis. All models were adjusted for age. A series of hierarchical logistic regression analyses were performed, evaluating whether each predictor was independently associated with the outcome variables. Multivariate models was then conducted, with gender being the first variable included in the models, followed by years at school, response outcome expectancies, work satisfaction, physical and mental work strain, and number of substantial SHC. Demographic variables were entered first into the model, which allowed for examination of the significance of hypothesized variables in predicting anxiety and/or depression, while controlling for demographic variables. Response outcome expectancies were then entered, to test the hypothesis that response outcome expectancies would predict anxiety and/or depression. In turn, work satisfaction, physical and mental work strain, and number of substantial SHC were entered in order to investigate if these variables would increase the prediction. The categorical work satisfaction variable with tree categories was recoded into a dichotomous variable, 0~“no” or “sometimes”, and 1~“yes”, before it was included in the models. The seven items measuring helplessness/hopelessness was computed into one variable ranging from 0 to 28, and a high score indicated a high degree of helplessness/hopelessness [36]. The two items measuring coping was computed into one variable ranging from 0 to 8, and a high score indicated a high degree of coping. The three continues variables measuring work satisfaction and physical and mental work strain were dichotomized using a median split (Table 2).

## Results

### Demographics

The demographic, work and psychological characteristics of the participating employees are shown in Tables 1 and 2.

**Table 1:** Mean and 95 % CI for person and health variables of the participants

Variables	Mean (95 % CI)
Age	44.1 (43.59–44.70)
Years of school	14.5 (14.39–14.68)
Coping (0–8)	6.03 (5.98–6.08)
Helplessness/hopelessness (0–28)	5.2 (4.99–5.40)
Number of substantial subjective health complaints (0–27)	3.26 (3.10–3.42)

**Table 2:** Percentage of person, anxiety, depression and work variables of the participants

Variables			%
Gender	Female		81.5
Comorbid anxiety and depression (n = 200)	Any level		11.6
		A little	7.9
		Some	3.0
		Severe	0.7
Anxiety (n = 61)	Any level		3.5
		A little	2.9
		Some	0.5
		Severe	0.1
Depression (n = 217)	Any level		12.6
		A little	10.4
		Some	1.7
		Severe	0.5
Do you enjoy your work?		Yes	89.6
		Sometimes	8.8
		No	0.4
Low work satisfaction			47.4
High physical work strain			40.3
High mental work strain			42.8

### Anxiety

Number of substantial SHC was the one variable that remained a significant factor in explaining anxiety among employees in the full model (see Table 3). The full model containing all predictors was statistically significant, X^2^ = 36.34 (10, N = 1570), *p* < .001, indicating that the model was able to distinguish between employees who did report anxiety and those who did not report anxiety (Nagelkerke’s R^2^ .14).

**Table 3:** Odds ratio and 95 % CI of person, work and psychological variables predicting likelihood of reporting severe anxiety and/or depression in the last 30 days

	Age-adjusted OR	Adjusted for yrs at school	Adjusted for outcome exp.	Adjusted for work satisfaction	Adjusted for work strain	Adjusted for # severe SHC
Anxiety, n = 31						
Age						
Women	0.54 (0.25–1.19)	0.57 (0.24–1.28)	0.60 (0.26–1.38)	0.61 (0.26–1.41)	0.60 (0.26–1.41)	0.44 (0.18–1.08)
Years of education	0.98 (0.86–1.11)	0.98 (0.87–1.11)	1.01 (0.88–1.14)	1.00 (0.88–1.13)	0.98 (0.86–1.12)	1.00 (0.87–1.15)
High helplessness/hopelessness	1.12 (1.04–1.21)*		1.08 (0.99–1.18)	1.06 (0.97–1.17)	1.07 (0.97–1.17)	1.02 (0.93–1.12)
Low coping	1.36 (1.00–1.84)*		1.21 (0.84–1.73)	1.15 (0.80–1.65)	1.14 (0.80–1.65)	1.16 (0.81–1.66)
Do not/sometimes enjoy work	2.34 (0.95–5.81)*			1.55 (0.55–4.34)	1.42 (0.50–4.08)	1.29 (0.44–3.77)
Low work satisfaction	1.19 (1.02–1.40)*			1.14 (0.95–1.36)	1.11 (0.91–1.34)	1.10 (0.91–1.33)
High physical work strain	1.08 (0.94–1.24)				0.94 (0.79–1.12)	0.93 (0.78–1.10)
High mental work strain	1.17 (1.02–1.35)*				1.11 (0.94–1.31)	1.05 (0.89–1.24)
Number of substantial subjective health complaints	1.26 (1.17–1.37)**					1.26 (1.14–1.38)**
Depression, n = 72						
Age						
Women	0.88 (0.49–1.58)	0.83 (0.46–1.50)	1.05 (0.56–1.95)	1.06 (0.57–1.99)	1.02 (0.54–1.92)	0.79 (0.41–1.53)
Years of education	1.01 (0.94–1.10)	1.01 (0.93–1.09)	1.07 (0.98–1.17)	1.07 (0.97–1.16)	1.04 (0.95–1.14)	1.05 (0.95–1.16)
High helplessness/hopelessness	1.24 (1.18–1.31)**		1.24 (1.17–1.31)**	1.22 (1.15–1.30)**	1.22 (1.15–1.30)**	1.19 (1.12–1.27)**
Low coping	1.49 (1.22–1.82)**		1.13 (0.89–1.45)	1.07 (0.84–1.37)	1.07 (0.83–1.37)	1.06 (0.82–1.37)
Do not/sometimes enjoy work	3.44 (1.97–6.03)**			1.43 (0.73–2.81)	1.23 (0.61–2.47)	1.01 (0.50–2.11)
Low work satisfaction	1.24 (1.11–1.37)**			1.14 (1.01–1.29)*	1.11 (0.98–1.26)	1.10 (0.97–1.26)
High physical work strain	1.12 (1.02–1.23)*				0.95 (0.84–1.06)	0.94 (0.83–1.05)
High mental work strain	1.25 (1.14–1.33)**				1.14 (1.02–1.28)*	1.09 (0.97–1.22)
Number of substantial subjective health complaints	1.27 (1.20–1.35)**					1.22 (1.14–1.31)**
Anxiety or depression, n = 103						
Age						
Women	0.74 (0.46–1.19)	0.73 (0.45–1.18)	0.85 (0.51–1.42)	0.86 (0.51–1.44)	0.84 (0.50–1.41)	0.61 (0.35–1.05)
Years of education	1.00 (0.94–1.08)	1.00 (0.93–1.07)	1.05 (0.98–1.13)	1.04 (0.97–1.13)	1.02 (0.94–1.10)	1.04 (0.96–1.13)
High helplessness/hopelessness	1.21 (1.16–1.27)**		1.20 (1.14–1.26)**	1.18 (1.12–1.24)**	1.18 (1.12–1.25)**	1.16 (1.10–1.22)**
Low coping	1.47 (1.24–1.75)**		1.16 (0.94–1.42)	1.10 (0.89–1.35)	1.09 (0.88–1.35)	1.08 (0.87–1.35)
Do not/sometimes enjoy work	3.21 (1.96–5.23)**			1.53 (0.86–2.72)	1.34 (0.74–2.42)	1.09 (0.58–2.06)
Low work satisfaction	1.23 (1.13–1.35)**			1.14 (1.03–1.27)*	1.11 (1.00–1.24)	1.11 (1.00–1.25)
High physical work strain	1.11 (1.03–1.20)*				0.94 (0.87–1.04)	0.93 (0.84–1.03)
High mental work strain	1.24 (1.14–1.34)**				1.14 (1.04–1.25)*	1.08 (0.98–1.19)
Number of substantial subjective health complaints	1.30 (1.23–1.37)**					1.26 (1.19–1.34)**
Comorbid anxiety and depression, n = 54						
Age						
Women	1.81 (0.77–4.28)	2.07 (0.81–5.27)	2.60 (0.96–7.04)	2.94 (1.06–8.17)*	2.74 (0.97–7.69)	1.84 (0.60–5.61)
Years of education	0.96 (0.88–1.05)	0.96 (0.88–1.06)	1.05 (0.95–1.16)	1.03 (0.93–1.15)	0.99 (0.88–1.10)	0.99 (0.88–1.13)
High helplessness/hopelessness	1.35 (1.26–1.44)**		1.35 (1.26–1.45)**	1.34 (1.25–1.45)**	1.34 (1.24–1.45)**	1.28 (1.18–1.39)**
Low coping	1.80 (1.46–2.23)**		1.30 (1.00–1.68)*	1.15 (0.88–1.50)	1.15 (0.87–1.50)	1.15 (0.86–1.53)
Do not/sometimes enjoy work	5.74 (3.19–10.31)**			2.48 (1.18–5.18)*	1.80 (0.83–3.90)	1.73 (0.73–4.09)
Low work satisfaction	1.33 (1.17–1.50)**			1.16 (1.00–1.36)	1.12 (0.95–1.32)	1.10 (0.93–1.31)
High physical work strain	1.22 (1.09–1.35)**				0.96 (0.83–1.10)	0.93 (0.80–1.08)
High mental work strain	1.40 (1.25–1.56)**				1.27 (1.10–1.46)**	1.15 (1.00–1.34)
Number of substantial subjective health complaints	1.39 (1.30–1.48)**					1.31 (1.21–1.42)**
Anxiety and/or depression, n = 157						
Age						
Women	0.96 (0.63–1.45)	0.96 (0.63–1.49)	1.17 (0.73–1.86)	1.20 (0.75–1.94)	1.16 (0.71–1.87)	0.81 (0.48–1.35)
Years of education	0.99 (0.93–1.04)	0.99 (0.93–1.04)	1.05 (0.99–1.12)	1.03 (0.97–1.11)	1.01 (0.95–1.08)	1.03 (0.96–1.11)
High helplessness/hopelessness	1.25 (1.21–1.31)**		1.24 (1.19–1.30)**	1.22 (0.93–1.33)**	1.22 (1.17–1.27)**	1.18 (1.13–1.24)**
Low coping	1.59 (1.38–1.83)**		1.18 (1.00–1.41)	1.11 (0.93–1.33)	1.12 (0.94–1.34)	1.11 (0.92–1.34)
Do not/sometimes enjoy work	3.99 (2.69–5.91)**			1.71 (1.06–2.77)*	1.43 (0.87–2.36)	1.18 (0.68–2.04)
Low work satisfaction	1.26 (1.17–1.36)**			1.15 (1.05–1.25)*	1.10 (1.01–1.21)*	1.10 (1.00–1.23)
High physical work strain	1.14 (1.07–1.22)**				0.96 (0.87–1.04)	0.95 (0.87–1.03)
High mental work strain	1.29 (1.20–1.37)**				1.17 (1.08–1.27)**	1.09 (1.00–1.19)*
Number of substantial subjective health complaints	1.34 (1.23–1.40)**					1.28 (1.22–1.35)**

* *p* ≤ .05, ** *p* ≤ .001

### Depression

Number of substantial SHC and helplessness/hopelessness were the two variables that remained significant factors in explaining depression among employees in the full model (see Table 3). Number of SHC was the variable with the highest explanatory power. The full model containing all predictors was statistically significant, X^2^ = 113.64 (10, N = 1575), *p* < .001, indicating that the model was able to distinguish between employees who did report depression and those who did not report depression (Nagelkerke’s R^2^ .23).

### Anxiety or Depression

Number of substantial SHC and helplessness/hopelessness were the two variables that remained significant factors in explaining anxiety or depression among employees in the full model (see Table 3). Number of SHC was the variable with the highest explanatory power. The full model containing all predictors was statistically significant, X^2^ = 147.02 (10, N = 1576), *p* < .001, indicating that the model was able to distinguish between employees who did report anxiety or depression and those who did not report anxiety or depression (Nagelkerke’s R^2^ .24).

### Comorbid Anxiety and Depression

Number of substantial SHC and helplessness/hopelessness were the two variables that remained significant factors in explaining comorbid anxiety and depression among employees in the full model (see Table 3). Number of SHC was the variable with the highest explanatory power. The full model containing all predictors was statistically significant, X^2^ = 168.16 (10, N = 1530), *p* < .001, indicating that the model was able to distinguish between employees who did report comorbid anxiety and depression and those who did not report comorbid anxiety and depression (Nagelkerke’s R^2^ .42).

### Anxiety and/or Depression

Number of substantial SHC, helplessness/hopelessness, and high mental work strain were the three variables that remained significant factors in explaining anxiety and/or depression among employees in the full model (see Table 3). Number of SHC was the variable with the highest explanatory power. The full model containing all predictors was statistically significant, X^2^ = 268.62 (10, N = 1626), *p* < .001, indicating that the model was able to distinguish between employees who did report anxiety and/or depression and those who did not report anxiety and/or depression (Nagelkerke’s R^2^ .34).

## Discussion

The aim of this study was to explore the association between anxiety and/or depression, and response outcome expectancies, work satisfaction, physical and mental work strain, and number of SHC in Norwegian municipal employees. The respondents in this sample reported on average a high degree of coping and a low degree of helplessness/hopelessness, which is to be expected in a healthy working population [35]. We hypothesized that response outcome expectancies would be the strongest predictor. The strongest association was however found between a high number of SHC and substantial anxiety and depression. A high degree of helplessness/hopelessness was a significant factor in explaining substantial depression, but not substantial anxiety. Thus, it may be that the depression-item has a higher explanatory power to the effect of helplessness/hopelessness in the analyses including both anxiety and depression as the dependent variable. The model with the highest proportion of variance accounted for was the one using comorbid anxiety and depression as dependent variable. According to Nagelkerke “pseudo” R^2^ the explained variance for this model was 41 %. For anxiety and depression alone the explained variance was lower, respectively 14 and 23 %.

Our findings are in accordance with a previous study that found a higher prevalence of SHC in groups that reported low coping in the normal working population, suggesting that lack of coping with stress, meaning low expectancies of a positive outcome, play an important role for normal SHC [20]. It may not be possible to prevent the occurrence of SHC. These complaints seem to be inherent in human nature and a part of everyday life, regardless of society or modern civilization [37]. However, it may be possible to influence employees’ response outcome expectancies, which in turn may influence the perception of health and further prevent negative consequences of such complaints [32]. Inability to cope with health complaints, the stress of an adverse work environment, or general life demands, may aggravate and reinforce the perception of health complaints, which in turn may have an effect on sensitization processes [38]. When complaints get intolerable we seek help and comfort, and this is the major reason for visiting the general practitioner [39]. Few of these patients have any serious medical condition or pathological findings, and there is no specific treatment for most of them. Despite this fact, and because the complaints are still very troublesome, many keep asking for medical explanations and medical help. A constant pursuit of answers and treatment for these conditions may have an unfavorable effect on the individual, such as unnecessary worrying [40]. Health worry has been found to predict the occurrence of health complaints [41], and both rumination and worry are central factors in anxiety disorders and depression [42]. A high frequency of visits to medical practitioners for symptoms that disrupt normal activities is also found to be a strong predictor for the development of medically unexplained physical symptoms [43]. There is a high focus on treatment for SHC, and many possible different treatment options, but little information about the limited effect many of the treatments have on these conditions. The strain on health from treatments that does not work is an important aspect to consider.

In this present study no and negative response outcome expectancies are a stronger predictor for anxiety and depression than physical and mental work strain. These results can be explained within the framework of CATS [18], where the expectancy of being able to cope with challenges or demands are more important for employees health than the physical demand itself. All stress stimuli are filtered before it gets access to the response system, and how a person reacts to the stimulus is determined by his or her experience of the demand and the expectancy of the outcome. If an employee expects to be able to handle a situation or demand with a positive result, the increase in activation is short and has a positive influence on health. If an employee expects not to cope with a situation or a demand, the activation may be sustained over time, which is associated with illness, disease, and poor health [44]. Our results also indicate that a feeling of helplessness (no response outcome expectancy) and hopelessness (negative response outcome expectancy), which both are proposed models for anxiety and depression [18, 45], are more important for employees’ mental health than work satisfaction.

Although the results were statistically significant, the effect sizes were relatively small. This may be a consequence of the large sample, as large samples make it more likely to achieve statistical significance even with small effect sizes. However, a large sample increases the likelihood that the results are in accordance with the actual population value, and even small effect sizes might have important practical significance [46]. Anxiety and depression have a substantially higher explanatory power in functional status than other SHC [29], and are among the most frequent causes of long-term sick leave and disability pensions in Norway [47]. Because the economic impact of sick leave is large, even marginal reductions and improvements may induce considerable savings. As response outcome expectancies may be possible to alter, our results imply that influencing employees response outcome expectancies could be an important focus in future workplace interventions targeting anxiety and depression. Nevertheless, it is probably equally important to also focus on creating an including work culture at the workplace, where employees with complaints are regarded as a part of the normal work environment and not excluded because of their health challenges.

### Strengths and Limitations

One of the main strengths of the study is that it is based on a large and representative sample of Norwegian municipality employees, which provides a good basis for generalization of the results to other worksites in the public sector. The sample is diverse with regard to work type and workplace size, which reduces the possibility of localization or group specific effects. However, we should be cautious about generalizing our finding to employees in the private sector.

A response rate of about 50 % may limit the validity of the findings. Even though considerable efforts were made to improve the response rate by providing information to the employees about the project, it remained low. The high predominance of women in the sample (81 %) is in accordance with the gender distribution of public sector employees, as about 70 % of all public sector employees are women, with the majority working in the municipalities [48]. In the two participating municipalities, 79 % and 68 % of the employees are women.

There might be limitations with using single-item questions when measuring psychological constructs [49] and the inclusion of validated scales on work satisfaction and work strain could provide more reliable conclusions regarding the relationship between anxiety, depression, and work characteristics. However, single-item questions measuring both work satisfaction [49] and work strain [50] indicates convergent validity with multi-item scales, which support the argument that a single-item question is acceptable. The anxiety- and depression items in SHC is found to be a good indicator in identifying anxiety and depression, when compared with widely used screening questionnaires [34]. From an ethical point of view, using a single-item question, as opposed to a multi-item scale, decreases the burden on the study participants.

## Conclusion

A high number of SHC, and a high degree of no and/or negative response outcome expectancies were associated with anxiety and depression in Norwegian municipal employees. The associations were small, although statistically significant. Because SHC seems difficult to prevent, we suggest that future workplace intervention targeting anxiety and depression could focus on influencing and altering employees’ response outcome expectancies, which may influence the perception of health and prevent negative consequences of SHC. However, we do need more research to investigate the relationship between response outcome expectancies and SHC in employees.
